# Optimal outpatient training for resident physicians’ general medicine in-training examination score: a cross-sectional study

**DOI:** 10.1186/s12909-025-06670-5

**Published:** 2025-01-11

**Authors:** Taiju Miyagami, Yuji Nishizaki, Taro Shimizu, Yu Yamamoto, Kiyoshi Shikino, Koshi Kataoka, Masanori Nojima, Gautam Deshpande, Toshio Naito, Yasuharu Tokuda

**Affiliations:** 1 Department of General Medicine, Faculty of Medicine, Juntendo University, Tokyo, Japan; 2 Division of Medical Education, Juntendo University School of Medicine, 2-1-1Bunkyo-Ku, HongoTokyo, Japan; 3 Department of Diagnostic and Generalist Medicine, Dokkyo Medical University Hospital, Tochigi, Japan; 4 Division of General Medicine, Center for Community Medicine, Jichi Medical University, Tochigi, Japan; 5 Department of General Medicine, Chiba University Hospital, Chiba, Japan; 6 Department of Community-Oriented Medical Education, Chiba University Graduate School of Medicine, Chiba, Japan; 7 Center for Translational Research, The Institute of Medical Science, The University of Tokyo, Tokyo, Japan; 8 Muribushi Okinawa Center for Teaching Hospitals, Okinawa, Japan; 9 Tokyo Foundation for Policy Research, Tokyo, Japan

**Keywords:** Outpatient training, Resident physicians, Clinical competence, Cross-sectional study

## Abstract

**Background:**

Outpatient training for resident physicians has been attracting attention in recent years. However, to our knowledge, there have only been a few surveys on outpatient training, particularly in Japan. This study evaluates outpatient care among Japanese resident physicians by determining how the volume of outpatient encounters and length of outpatient training correlate with residents’ clinical competence.

**Methods:**

This study utilised the results of the General Medicine In-Training Examination (GM-ITE; resident clinical competency assessment) for 2,554 post-graduate year 2 (PGY 2) resident physicians in Japan, as well as a self-reported questionnaire regarding their educational training environments conducted after the examination. We investigated whether GM-ITE scores correlated with daily outpatient volume and duration of outpatient training.

**Results:**

Regarding outpatient volume, having 1–5 new patient encounters per day was significantly associated with higher GM-ITE scores by multilevel analysis [0 patients: average score 43.7, 1–5 patients: adjusted estimated coefficient (aEC) 1.99, 95% confidence interval (CI) 0.44 to 3.55, *P* = 0.01]. Regarding the duration of outpatient training, residents trained for one month had the highest GM-ITE scores (one month: average score 46.9; two months: aEC -1.44, 95% CI -2.29 to -0.60, *P* < 0.001; three months: aEC -1.44, 95% CI -2.22 to -0.65, *P* < 0.001).

**Conclusion:**

Minimal daily new outpatient visits and one month of outpatient training effectively correlated with residents’ basic clinical competence.

**Trial registration:**

This study was approved by the Ethics Committee of the Japan Institute for Advancement of Medical Education Program (JAMEP; No. 22–30) and retrospectively registered.

**Supplementary Information:**

The online version contains supplementary material available at 10.1186/s12909-025-06670-5.

## Background

Residency training has a common goal worldwide: to help resident physicians become competent and independent physicians who can provide safe and effective care to their patients in a variety of settings and contexts. Resident physicians’ work can be broadly divided into outpatient and inpatient care. It has been reported that residents’ main work is primarily in the inpatient care setting [1, 2]. This has led to a tendency for most internal medicine residents to minimise outpatient care [3]. Against this background, U.S. internal medicine residents have received little training in chronic disease, leading to poor quality chronic disease care and patient dissatisfaction [4, 5]. U.S. hospitals and clinics minimise outpatient training as it may not be cost-effective to have young residents treat outpatients [6–8]. Other studies have identified the loss of time (i.e. fewer patients available for care and longer clinic hours) due to student education during outpatient visits as a concern [9].

Nonetheless, it has been reported that clinical training in outpatient settings is needed in the U.S. due to the recent trend toward fewer inpatient admissions and shorter lengths of stay [10, 11]. Additionally, clinical training in outpatient settings is becoming increasingly important to gain experience with asthma and human immunodeficiency virus related diseases, which, in recent years, have become more common in outpatient settings. Subsequently, steps are being taken in the U.S. to encourage ambulatory training and increase the number of trainees [12].

In Japan, the core model curriculum for medical students was revised in 2022 [13]. One of the basic policies is to revise the qualities and skills required of physicians in anticipation of society’s needs 20 years from now. Japan’s demographic structure, the so-called ‘2040 problem’, is expected to see continued declines in birth rate and increase in age. Thus, there will be an even greater need to deal with patients with multiple comorbidities and diseases. As the management of several diseases shifts from inpatient to outpatient care, and the healthcare delivery system evolves to include community-based integrated care, the importance of general outpatient care is increasing. Furthermore, the ability to make accurate clinical judgments for patients who have not yet been diagnosed is becoming increasingly critical, and outpatient care is expected to play a central role in addressing these challenges. The core model curriculum for medical schools emphasizes the importance of taking a comprehensive approach to patient care. In particular, through cross-disciplinary care, outpatient training enables residents to learn patient-centred medicine, adopt a "patient-centred approach," and understand the social backgrounds of patients. This kind of education also helps residents grasp the realities and challenges of their local community and acquire the knowledge and skills necessary for practicing primary care. In light of these considerations, general outpatient training has been a compulsory part of postgraduate clinical training since the 2020 revision of the Ministry of Health, Labour and Welfare’s (MHLW) guidelines [14]. This training is designed to ensure that doctors gain a wide range of experience, including the treatment of new patients and the ongoing care of chronic diseases, without being limited to specific symptoms or diseases. Resident physicians are expected to be able to diagnose and treat frequently occurring syndromes and conditions through an appropriate clinical reasoning process and provide ongoing care for major chronic diseases [14].

Despite these expectations, no previous studies have evaluated the relationship between basic clinical competence and the optimal number of patients that a resident physician should see in the outpatient clinic. We hypothesised that greater daily numbers of patients seen during outpatient training would manifest greater improvements in knowledge-based competence of resident physicians. We used the General Medicine In-Training Examination (GM-ITE) to evaluate the relationship between knowledge-based competence of resident physicians and the appropriate number and duration of outpatients [15].

## Methods

### Design and participants

This study used a survey to conduct a cross-sectional examination of resident physicians in Japan. Beginning in 2004, Japan required all newly graduated residents to participate in a compulsory, two-year multidisciplinary training program after graduation. This program encompasses rotations across various medical fields, including internal medicine, emergency care, paediatrics, gynaecology, psychiatry, surgery, and community health, assessing students’ competency in the national medical licensure exam taken during their last year of medical school. In the fiscal year 2022, there were a total of 18,655 resident physicians in Japan. Both post-graduate year 1 (PGY 1) and post-graduate year 2 (PGY 2) resident physicians take the same GM-ITE at each hospital on a voluntary basis. Immediately following this examination, they completed self-reported questionnaires that aimed to collect comprehensive data on their current working conditions. The GM-ITE was conducted from 17 to 30 January 2023. This study was conducted in accordance with the STROBE (Strengthening the Reporting of Observational Studies in Epidemiology) guidelines [16].

### Variables

The GM-ITE assesses general clinical knowledge and practical relevance in accordance with the MHLW guidelines for Clinical Training [17]. The exam provides feedback on individual residents and evaluates training programs and institutions. The GM-ITE is a computer-based testing (CBT) that consists of 80 multiple-choice questions with optional tests. In 2022, 48.3% of all resident physicians in Japan took it.

In line with the Japanese MHLW goals for resident physicians, the 2022 GM-ITE covers basic clinical knowledge, including 1) medical interviewing and professionalism, 2) symptomatology and clinical reasoning, 3) physical examination and clinical procedures, and 4) disease knowledge. Baseline characteristics including resident physicians’ age, gender, hospital location (urban or rural; the 20 cities designated by the Ministry of Internal Affairs and Communications and the 23 wards of Tokyo were defined as urban areas, and all other cities were defined as rural areas), general hospital type (community-based or university hospital), self-study time per day, duty hours per week, general outpatient training period excluding emergency outpatient services, average daily number of new and follow-up outpatients seen during general outpatient training, and style of outpatient training were evaluated. As demonstrated in Supplemental Digital Appendix 1, the outpatient training style was divided into block (one month of training only in the outpatient department), parallel (one month of training in the outpatient department while treating ward patients), and mixed styles (using both block and parallel training depending on the duration of training). The PGY 1 was excluded to account for the possibility that some individuals may not have had outpatient training, and GM-ITE was timed to approximate the completion of training in the fiscal year. The outcomes were evaluated in relation to the duration of outpatient training, the number of new and follow-up outpatients and their GM-ITE scores.

### Ethics

This study was approved by the Ethics Committee of the Japan Institute for Advancement of Medical Education Program (JAMEP; No. 22–30) and conducted in accordance with the principles of the Declaration of Helsinki. All participants reviewed the study document detailing data anonymisation, voluntary participation, and the dissemination of research results prior to participation. Only participants who provided informed consent (opt-in) were included in the study. Additionally, the participants could withdraw at any time.

### Statistics

The results are presented as means ± standard deviation for continuous variables or as prevalence (%) for categorical variables. If there were any unanswered questions for any of the measurement items, they were all excluded. A chi-squared test was performed, stratified by the number of new outpatients and follow-up outpatients. Multilevel analysis was performed to evaluate the relationship among outpatient numbers (0, 1–5, 6 or more), outpatient training period, and GM-ITE scores. The other covariates were sex, hospital location, hospital type, self-study time per day, duty hours, and outpatient training style. All statistical analyses were performed using SAS version 9.4 (SAS Institute, Cary, NC, USA). Statistical significance was set at *P* < 0.05.

## Results

**Fig. 1:**
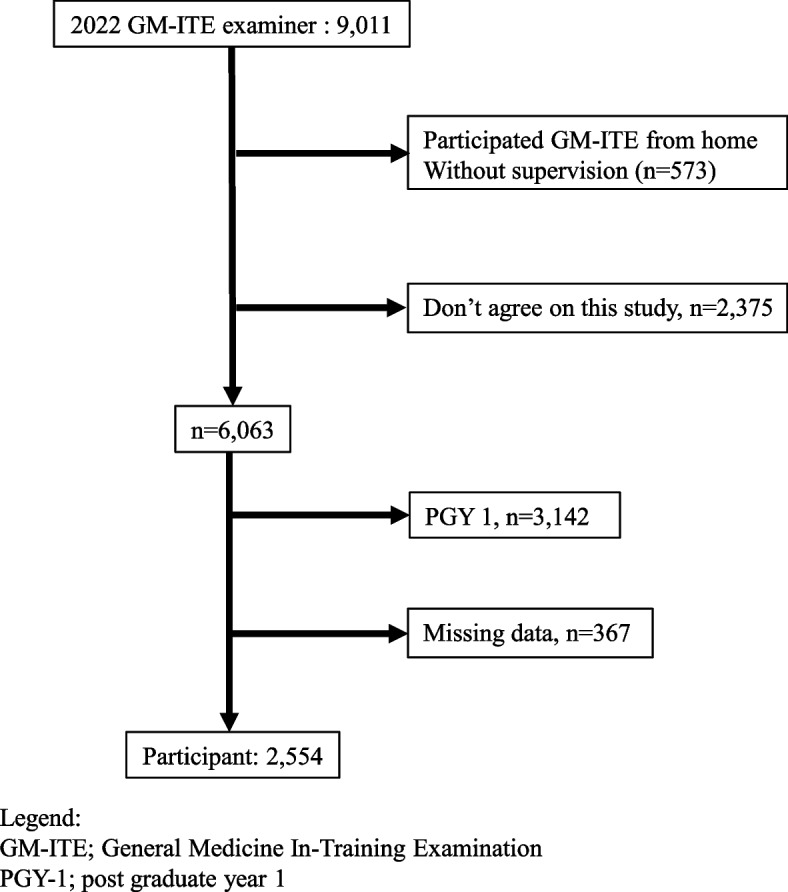
Participants flow

A total of 9,011 residents from 662 teaching hospitals participated in the 2022 GM-ITE. Participants who were examined through GM-ITE from home without supervision (*n* = 573), did not provide consent (*n* = 2,375), were in their PGY 1 (*n* = 3,142), or had at least one missing data variable (*n* = 367) were excluded. Thus, 2,554 participants were included in the final analysis (Fig. 1).

In total, 802 (31.4%) participants were female, 1,705 (66.8%) were trained in rural areas, and 2,096 (82.1%) were located in community-based hospitals. Resident physicians reporting 0–30 min and 31–60 min of self-study time per day were approximately equal at 1,071 (41.9%) and 1,027 (40.2%), respectively. The most common number of duty hours per week was ≤ 59 h per week, reported by 1,291 (50.5%) resident physicians.

**Fig. 2:**
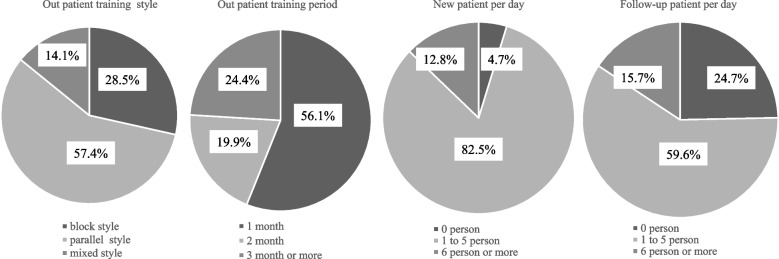
The actual state of outpatient training for resident physicians

The most common outpatient training style was parallel training, used by 1,465 residents (57.4%). The most common outpatient training period was one month 1,432 (56.1%). Seeing 1–5 new outpatients per day and seeing 1–5 follow-up outpatients per day was the most common, with 2,106 (82.5%) and 1,523 participants (59.6%), respectively (Fig. 2). Further comparisons are made concerning university hospitals and community-based hospitals, respectively (please see Supplemental Digital Appendix 2).

The number of new outpatients seen per day was divided into three groups: 0, 1–5, and 6 or more. Significant differences among these groups were observed in hospital type, self-study time, outpatient training period, and outpatient training style. Specifically, community-based hospitals tended to have more residents in the 1–5 patient group compared to university hospitals. The duration of self-study was longer in the 1–5 and 6 or more patient groups than in the 0 patient group. Additionally, the duration of outpatient training increased with the number of patients. Regarding training style, the block style was associated with more residents in the 6 or more groups, while the mixed style had more residents in the 0 patient group compared to the parallel group. GM-IE scores were highest in the 1–5 patient group (Table 1).

**Table 1:** Stratified by number of new outpatients

	All *n* = 2554	(%) or SD	0 persons *n* = 121 (4.7%)	(%) or SD	1 to 5 persons *n* = 2106 (82.5%)	(%) or SD	6 persons or more *n* = 327 (12.8%)	(%) or SD	*P* value
**Sex**									0.252
Female	802	31.4	35	28.9	676	32.1	91	27.8	
Male	1752	68.6	86	71.1	1430	67.9	236	72.2	
**Hospital location**									0.615
Rural	1705	66.8	83	68.6	1397	66.3	225	68.8	
Urban	849	33.2	38	31.4	709	33.7	102	31.2	
**Hospital type**									0.045
Community-based hospital	2096	82.1	91	75.2	1745	82.9	260	79.5	
University hospital	458	17.9	30	24.8	361	17.1	67	20.5	
**Self-study time per day**									< .001
None	48	1.9	8	6.6	37	1.76	3	0.9	
0–30 min	1071	41.9	63	52.1	864	41	144	44.1	
31–60 min	1027	40.2	42	34.7	861	40.9	124	37.9	
61–90 min	320	12.5	7	5.8	271	12.9	42	12.8	
91 min or more	88	3.5	1	0.8	73	3.5	14	4.3	
**Duty hour**									0.104
0–59 h per week	1291	50.5	68	56.2	1054	50.1	169	51.7	
60–79 h per week	880	34.5	38	31.4	745	35.4	97	29.7	
> 80 h per week	383	15.0	15	12.4	307	14.6	61	18.7	
**Outpatient training period**									< .001
1 month	1432	56.1	74	61.2	1215	57.7	143	43.7	
2 months	508	19.9	23	19	408	19.4	77	23.6	
3 months or more	614	24.0	24	19.8	483	22.9	107	32.7	
**Outpatient training style**									0.021
block style	728	28.5	25	20.7	607	28.8	96	29.4	
parallel style	1465	57.4	69	57.0	1219	57.9	177	54.1	
mixed style	361	14.1	27	22.3	280	13.3	54	16.5	
**GM-ITE Total Score**	46.3	8.7	43.7	9.1	46.7	8.5	44.2	9.0	< .001

Nominal variables are shown as n(%) and continuous variables are shown as Mean ± SD

*GM-ITE* General Medicine In-Training Examination. Only GM-ITE is expressed as Mean ± SD

The number of follow-up outpatients per day was also divided into three groups: 0, 1–5, and 6 or more. Significant differences were found among these groups across all items. Female and community-based hospitals tended to see fewer follow-up outpatients, while rural hospital locations tended to see more follow-up patients. The longer the self-study time, the more follow-up outpatients were seen. Similarly, a longer outpatient training period correlated with more follow-up patients. In terms of training style, the block style tended to see fewer follow-up outpatients compared to the parallel style. The GM-ITE score was highest in the 1–5 patient group (Table 2).

**Table 2:** Stratified by number of follow-up outpatients

	All *n* = 2554	(%) or SD	0 persons *n* = 630 (24.7%)	(%) or SD	1 to 5 persons *n* = 1523 (59.6%)	(%) or SD	6 persons or more *n* = 401 (15.7%)	(%) or SD	*P* value
**Sex**									< .001
Female	802	31.4	242	38.4	459	30.1	101	25.2	
Male	1752	68.6	388	61.6	1064	69.9	300	74.8	
**Hospital location**									< .001
Rural	1705	66.8	377	59.8	1048	68.8	280	69.8	
Urban	849	33.2	253	40.2	475	31.2	121	30.2	
**Hospital type**									0.001
Community-based hospital	2096	82.1	544	86.4	1240	81.4	312	77.8	
University hospital	458	17.9	86	13.7	283	18.6	89	22.2	
**Self-study time per day**									< .001
None	48	1.9	21	3.3	20	1.3	7	1.8	
0–30 min	1071	41.9	287	45.6	614	40.3	170	42.4	
31–60 min	1027	40.2	244	38.7	633	41.6	150	37.4	
61–90 min	320	12.5	67	10.6	203	13.3	50	12.5	
91 min or more	88	3.5	11	1.8	53	3.5	24	6.0	
**Duty hour**									0.009
0–59 h per week	1291	50.5	342	54.3	764	50.2	185	46.1	
60–79 h per week	880	34.5	204	32.4	541	35.5	135	33.7	
> 80 h per week	383	15.0	84	13.3	218	14.3	81	20.2	
**Outpatient training period**									0.005
1 month	1432	56.1	392	62.2	834	54.8	206	51.4	
2 months	508	19.9	102	16.2	317	20.8	89	22.2	
3 months or more	614	24.0	136	21.6	372	24.4	106	26.4	
**Outpatient training style**									< .001
block style	728	28.5	129	20.5	467	30.7	132	32.9	
parallel style	1465	57.4	411	65.2	847	55.6	207	51.6	
mixed style	361	14.1	90	14.3	209	13.7	62	15.5	
**GM-ITE Total Score**	46.3	8.7	46.1	8.3	46.6	8.8	45.2	9.0	0.012

Nominal variables are shown as n(%), and continuous variables are shown as Mean ± SD

*GM-ITE* General Medicine In-Training Examination. Only GM-ITE is expressed as Mean ± SD

A multilevel analysis was performed using the GM-ITE score as the objective variable. Regarding the number of new outpatient visits per day, having 1–5 patients was significantly associated with higher GM-ITE scores [0 patients: average score 43.7, 1–5 patients: adjusted estimated coefficient (aEC) 1.99, 95% confidence interval (CI) 0.44 to 3.55, *P* = 0.012]. There was no significant difference in follow-up patients. Concerning the duration of outpatient training, residents who rotated for one month had the highest GM-ITE scores (one month: average score 46.9; two months: aEC −1.44, 95% CI −2.29 to −0.60, *P* < 0.001; three months: aEC −1.44, 95% CI −2.22 to −0.65, *P* = 0.007; see Table 3).

**Table 3:** Univariate/multivariate analysis with GM-ITE total score as the objective variable

		Univariable				Multilevel		
		95% CI				95% CI		
	Estimated coefficient	lower limit	upper limit	*P* value	Adjusted estimated coefficient	lower limit	upper limit	*P* value
**Sex**								
Female (vs Male)	−0.86	−1.58	−0.13	0.02	−0.68	−1.35	0.00	0.05
**Hospital location**								
Rural (vs Urban)	−0.18	−0.89	0.54	0.63	−0.30	−1.29	0.69	0.55
**Hospital type**								
Community-based hospital (vs University hospital)	5.30	4.45	6.16	< .001	3.67	2.20	5.13	< .001
**Self-study time per day**								
None	Ref				Ref			
0–30 min	2.10	−0.38	4.59	0.10	1.66	−0.64	3.96	0.16
31–60 min	4.16	1.68	6.65	< .001	2.99	0.68	5.30	< .001
61–90 min	4.76	2.15	7.37	< .001	3.36	0.94	5.78	< .001
91 min or more	6.35	3.33	9.38	< .001	4.53	1.71	7.35	< .001
**Duty hour**								
0–59 h per week	Ref				Ref			
60–79 h per week	2.59	1.85	3.32	< .001	1.24	0.54	1.93	< .001
> 80 h per week	2.79	1.81	3.76	< .001	1.27	0.31	2.22	< .001
**Outpatient training period**								
1 month	Ref				Ref			
2 months	−1.99	−2.87	−1.11	< .001	−1.44	−2.29	−0.60	< .001
3 months or more	−1.04	−1.86	−0.22	0.01	−1.44	−2.22	−0.65	< .001
**Outpatient training style**								
block style	Ref				Ref			
parallel style	−0.71	−1.48	0.06	0.07	0.18	−0.58	0.95	0.64
mixed style	−1.97	−2.96	−0.97	< .001	−1.29	−2.22	−0.35	< .001
**New outpatient per day**								
0 persons	Ref				Ref			
1 to 5 persons	2.99	1.40	4.57	< .001	1.99	0.44	3.55	0.01
6 persons or more	0.52	−1.28	2.32	0.57	0.36	−1.44	2.16	0.69
**Follow-up outpatient per day**								
0 persons	Ref				Ref			
1 to 5 persons	0.54	−0.27	1.35	0.19	0.11	−0.71	0.93	0.79
6 persons or more	−0.87	−1.96	0.21	0.12	−0.59	−1.72	0.55	0.31

*CI* confidence interval, *GM-ITE* General Medicine In-Training Examination. The GM-ITE score was analysed as the objective variable

## Discussion

In recent years, outpatient training for resident physicians has been attracting attention; however, there have only been a few surveys on outpatient training, especially in Japan. Therefore, we conducted a survey on the actual situation of outpatient training for resident physicians.

The results of this study showed that 1–5 new patients per day, one month of outpatient training, and the block training style scored the highest, with significant differences, which are discussed below.

### Relationship between the number of outpatients

Initially, we assumed that the higher the number of outpatients per day, the higher the basic clinical competence. Previous studies have shown that the mortality rate decreased with more experience in surgical procedures and cases requiring expertise, such as cancer [18, 19]. However, in a previous report examining the number of patients treated and outcomes of patients with common diseases such as pneumonia, heart failure, and myocardial infarction, up to a certain number of patients, the more patients were seen, the better the prognosis; when the number of patients increased too much, outcomes such as mortality rates did not improve [20]. In this study, GM-ITE scores were highest when residents saw 1–5 new outpatients per day, compared to seeing 6 or more new outpatients per day. Therefore, it is important to attempt to define an optimal number of patients seen in outpatient care by residents that correlate with GM-ITE scores, as shown in this study.

Additionally, a report on the number of hospitalised patients treated by residents in Japan showed that the group that saw the most patients had the highest score. This result differed from the present study [21]. This discrepancy could be attributed to the difference in time course between the inpatient and the outpatient clinic; the outpatient clinic generally requires disposition decision-making—to admit or not admit patients—on the same day. Further, it was considered that the time-course delays in the outpatient setting and the work process of having to appropriately handle patients with uncertain diagnoses were more complex than for inpatients; the associated competencies required of the outpatient resident may be more burdensome than those for inpatient care [22, 23].

Regarding outpatient clinic hours, the MHLW stipulates that resident physicians are expected to see 1–2 new outpatients per half-day and to conduct a medical interview and physical examination over 10–30 min, followed by consultation with the supervising physician [14]. This stratification was made based on the Japanese Guideline 2020 Statutory Clinical Training. The results of this study are consistent with the number of people treated as determined by the MHLW. Since 66.6% of Japanese specialists provide general outpatient care within 10 min per patient, the number of residents providing outpatient care is ‘negative’ in terms of hospital revenue [24]. Therefore, training in outpatient care in Japan is primarily for educational purposes.

The following reasons were considered for the lack of significant differences in the number of outpatient follow-up visits. In the U.S., continuing outpatient care in clinics was reported to be highly stressful for residents due to inadequate resources and also highly stressful for those teaching [25]. In Japan, continuing outpatient care is often provided in hospitals, and it is not clear to what extent this affected the results. Considering the content of the GM-ITE, the number of new outpatients might have had more influence than the number of follow-up visits since many of the questions were related to clinical reasoning and symptomatology, and few questions were asked about knowledge of chronic diseases, such as lifestyle-related diseases.

### Duration of outpatient training

As per the Japanese MHLW curriculum issued in the year 2020, it is mandatory to have at least 4 weeks of outpatient training [14]. In this study, the highest GM-ITE scores were seen for one month of training, generally the smallest block of outpatient training available to learners. In the Japanese residency system, the overall training period is limited to two years, and it may be important to provide training in a wide range of fields, not limited to outpatient training, to improve knowledge-based competence of resident physicians. A previous report noted that the appropriate burden of emergency duty and the number of rotations in general medicine were related to the resident’s clinical ability score, and even a one-month outpatient training period may be effective in improving the score [26]. However, the optimal length of outpatient training must be considered to improve knowledge-based competence of resident physicians as a whole.

### Training styles

The results of this study showed that mixed training had predominantly lower scores than block or parallel training. Previous reports have shown that one year of continuous outpatient training increases resident and patient satisfaction and improves the quality of care [27]. In the U.S., 1–2 weeks of outpatient care every 3–6 weeks became the norm [25]. Further, having one week of outpatient visits every four weeks increased resident satisfaction and learning opportunities [28].

An existing study created a 50/50 model, a month-to-month model with two outpatient clinic weeks per month and no outpatient clinic months at all, which allowed residents to see more patients and improve the quality of care compared to before the intervention [29]. These were evaluated through a longitudinal training style, which, in the context of this study, was parallel training. There are no reports that clearly evaluate the clinical effects of the differences between the block and parallel training styles; however, it is essential to see continuously patients over time [30]. Furthermore, training in which the instructor and learner have a vertical relationship is also effective for improving the learner's skills [31]. Outpatient training in Japan can be either exclusively outpatient (block training) or occasional outpatient training in between-ward care (parallel training). Nonetheless, there is no specific definition of the number or frequency of outpatient visits during parallel training. In the future, it is necessary to confirm the details of parallel training. In view of the foregoing, this study cannot definitively determine whether the block or parallel style is more superior. The mixed training group had smaller overall numbers than the block or parallel training groups. Against this background, we speculated that the mixed training groups would have lower scores. Here, the mixed training groups may not have been staffed primarily for resident education; rather, the hospitals assigned residents to outpatient departments or wards according to the needs and circumstances of each department.

### GM-ITE score

The GM-ITE score is not specialized for outpatient training clinical skills. Therefore, the authors (TM, YN, KS and TS) only examined and extracted the 17 items related to outpatient training. We reanalysed the data using these 17 items, and the results were the same as those using the 80 items (Supplemental Digital Appendix 3). Therefore, the items related to outpatient training may have affected the results of this study.

The results of this study showed that the scores of general based hospitals were higher than those of university hospitals. This has been discussed in previous reports that used GM-ITE. Compared to university hospitals, general hospitals tended to score higher in the physical examination and clinical procedures section [32]. This indicates that general hospitals are more focused on education. In addition, since the GM-ITE score is higher when self-study time and working hours are longer, motivation may be involved.

### Limitations

This study has several limitations. First, it does not specifically measure a more precise optimal number of patients seen within the 1–5 new outpatient variable. Further, the reason for the low scores for the mixed training remains unclear and was not evaluated in this study. The evaluation focused only on the external aspects of ambulatory training and did not evaluate qualitative aspects, such as types or amount of teaching in ambulatory training. Furthermore, there has been no exploration of background factors, such as the motivation of the residents or the department they wish to work in. Only about 25% of PGY 2 trainees nationwide participate in ambulatory training. For parallel training, we do not know how many times per week ambulatory training was performed. Additionally, because this is a cross-sectional study, causal relationships are speculative. In addition, GM-ITE does not evaluate residents’ performance in clinical situations; it only evaluates the knowledge-based part of clinical skills and does not cover all clinical skills. Of the GM-ITE score, the number of questions related to outpatient training knowledge-based clinical skills is not particularly high, at 17 out of 80 (21.3%). Furthermore, the GM-ITE score is likely to be significantly affected by motivation, as people who spend a lot of time studying or working get higher scores, while those who see no patients per day get lower scores.

## Conclusion

To the best of our knowledge, this is the first study to evaluate the relationships between resident physicians’ basic clinical competence, and the number of outpatients they see per day and the duration of outpatient training, respectively.

The results showed that even a small number of patients in the new outpatient clinic are associated with more competent performance in basic clinical skills examinations. The minimum duration of training in clinic visits, though just one month, was also found to be associated with better testing performance. Appropriate-volume outpatient training experience may represent effective strategies for the development of future clinical training curricula and programs. Future studies should evaluate not only the quantity but also the quality of training.

## Supplementary Information

Supplementary Material 1: Supplemental Digital Appendix 1. Outpatient training style of resident physician. Legend: This is just an example, but parallel training may or may not be done as an outpatient on some days. Block training is only offered at the outpatient clinic for one month. The mixed style changes from month to month, whether it is parallel training or block training.

Supplementary Material 2: Supplemental Digital Appendix 2. Outpatient Training Compared to Community based Hospitals and University Hospitals.

Supplementary Material 3.

## Data Availability

The data are unavailable due to participants’ non-consent for public data sharing.
