# Supplementary material for: Optimal outpatient training for resident physicians’ general medicine in-training examination score: a cross-sectional study
Source: BMC Med Educ. 2025 Jan 11;25:49. doi: 10.1186/s12909-025-06670-5 (PMC11724509; doi:10.1186/s12909-025-06670-5)
Supplement: Supplementary file 1 — Supplementary Material 1: Supplemental Digital Appendix 1. Outpatient training style of resident physician. Legend: This is just an example, but parallel training may or may not be done as an outpatient on some days. Block training is only offered at the outpatient clinic for one month. The mixed style changes from month to month, whether it is parallel training or block training. [file 12909_2025_6670_MOESM1_ESM.pdf]

Supplemental Digital Appendix 1: Outpatient training style of resident physician

Parallel Style

January		Monday	Tuesday	Wednesday	Thursday	Friday	February		Monday	Tuesday	Wednesday	Thursday	Friday
1 Week	AM	Inpatient	Inpatient	Outpatient	Inpatient	Inpatient	1 Week	AM	Inpatient	Inpatient	Outpatient	Inpatient	Inpatient
	PM	Outpatient	Inpatient	Inpatient	Inpatient	Inpatient		PM	Outpatient	Inpatient	Inpatient	Inpatient	Inpatient
2 Week	AM	Inpatient	Inpatient	Inpatient	Inpatient	Inpatient	2 Week	AM	Inpatient	Inpatient	Inpatient	Inpatient	Inpatient
	PM	Outpatient	Inpatient	Outpatient	Inpatient	Outpatient		PM	Outpatient	Inpatient	Outpatient	Inpatient	Outpatient
3 Week	AM	Inpatient	Inpatient	Outpatient	Inpatient	Inpatient	3 Week	AM	Inpatient	Inpatient	Outpatient	Inpatient	Inpatient
	PM	Outpatient	Inpatient	Inpatient	Outpatient	Outpatient		PM	Outpatient	Inpatient	Inpatient	Outpatient	Outpatient
4 Week	AM	Inpatient	Inpatient	Outpatient	Inpatient	Inpatient	4 Week	AM	Inpatient	Inpatient	Outpatient	Inpatient	Inpatient
	PM	Outpatient	Inpatient	Inpatient	Inpatient	Outpatient		PM	Outpatient	Inpatient	Inpatient	Inpatient	Outpatient

Block style

January		Monday	Tuesday	Wednesday	Thursday	Friday	February		Monday	Tuesday	Wednesday	Thursday	Friday
1 Week	AM	Inpatient	Inpatient	Inpatient	Inpatient	Inpatient	1 Week	AM	Outpatient	Outpatient	Outpatient	Outpatient	Outpatient
	PM	Inpatient	Inpatient	Inpatient	Inpatient	Inpatient		PM	Outpatient	Outpatient	Outpatient	Outpatient	Outpatient
2 Week	AM	Inpatient	Inpatient	Inpatient	Inpatient	Inpatient	2 Week	AM	Outpatient	Outpatient	Outpatient	Outpatient	Outpatient
	PM	Inpatient	Inpatient	Inpatient	Inpatient	Inpatient		PM	Outpatient	Outpatient	Outpatient	Outpatient	Outpatient
3 Week	AM	Inpatient	Inpatient	Inpatient	Inpatient	Inpatient	3 Week	AM	Outpatient	Outpatient	Outpatient	Outpatient	Outpatient
	PM	Inpatient	Inpatient	Inpatient	Inpatient	Inpatient		PM	Outpatient	Outpatient	Outpatient	Outpatient	Outpatient
4 Week	AM	Inpatient	Inpatient	Inpatient	Inpatient	Inpatient	4 Week	AM	Outpatient	Outpatient	Outpatient	Outpatient	Outpatient
	PM	Inpatient	Inpatient	Inpatient	Inpatient	Inpatient		PM	Outpatient	Outpatient	Outpatient	Outpatient	Outpatient

Mixed style

January		Monday	Tuesday	Wednesday	Thursday	Friday	February		Monday	Tuesday	Wednesday	Thursday	Friday
1 Week	AM	Inpatient	Inpatient	Outpatient	Inpatient	Inpatient	1 Week	AM	Outpatient	Outpatient	Outpatient	Outpatient	Outpatient
	PM	Outpatient	Inpatient	Inpatient	Inpatient	Inpatient		PM	Outpatient	Outpatient	Outpatient	Outpatient	Outpatient
2 Week	AM	Inpatient	Inpatient	Inpatient	Inpatient	Inpatient	2 Week	AM	Outpatient	Outpatient	Outpatient	Outpatient	Outpatient
	PM	Outpatient	Inpatient	Outpatient	Inpatient	Outpatient		PM	Outpatient	Outpatient	Outpatient	Outpatient	Outpatient
3 Week	AM	Inpatient	Inpatient	Outpatient	Inpatient	Inpatient	3 Week	AM	Outpatient	Outpatient	Outpatient	Outpatient	Outpatient
	PM	Outpatient	Inpatient	Inpatient	Outpatient	Outpatient		PM	Outpatient	Outpatient	Outpatient	Outpatient	Outpatient
4 Week	AM	Inpatient	Inpatient	Outpatient	Inpatient	Inpatient	4 Week	AM	Outpatient	Outpatient	Outpatient	Outpatient	Outpatient
	PM	Outpatient	Inpatient	Inpatient	Inpatient	Outpatient		PM	Outpatient	Outpatient	Outpatient	Outpatient	Outpatient

The above is just one example.
In parallel style training, you will work in the outpatient department and on the hospital ward at the same time over the course of a month.
In block style training, you will only work in the outpatient department for a month. In the month you work on the hospital ward, you will only work on the hospital ward.
In mixed style training, there are months where you will work in the outpatient department and on the hospital ward at the same time over the course of a month, as in parallel training, and months where you will only work in the outpatient department, as in block training.
