# Supplementary material for: Optimal outpatient training for resident physicians’ general medicine in-training examination score: a cross-sectional study
Source: BMC Med Educ. 2025 Jan 11;25:49. doi: 10.1186/s12909-025-06670-5 (PMC11724509; doi:10.1186/s12909-025-06670-5)

Supplemental Digital Appendix 2: Outpatient Training Compared to Community-based Hospitals and University Hospitals

Community-based hospital

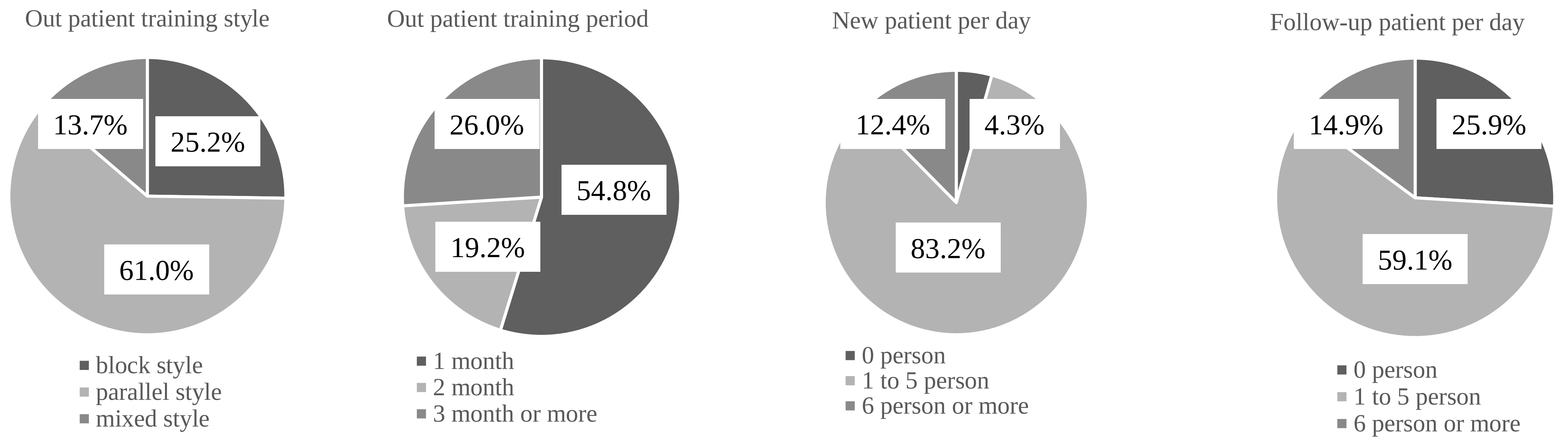

University hospital

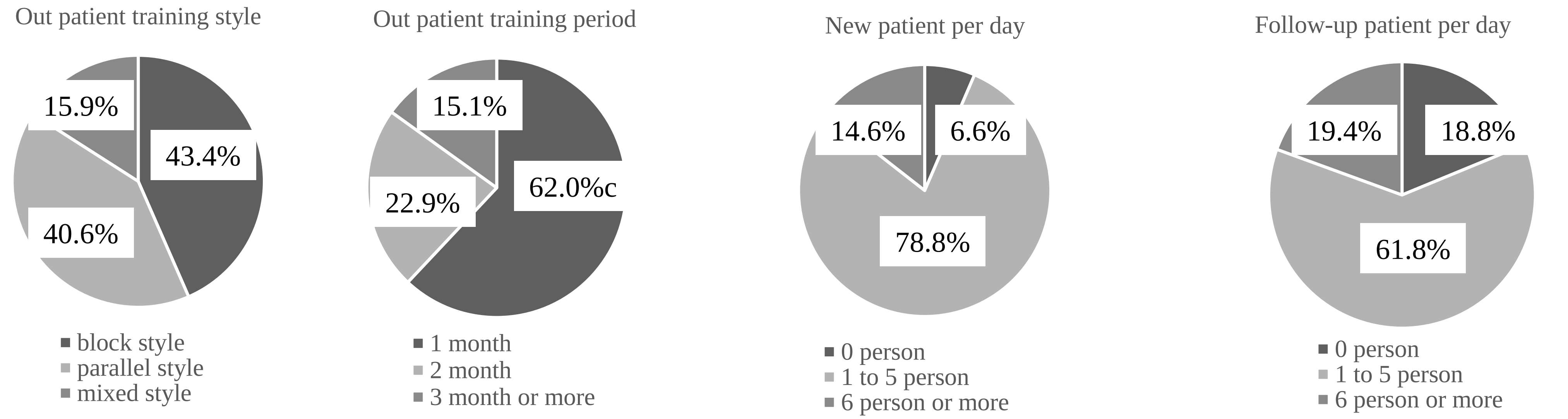

Supplement: Supplementary file 2 — Supplementary Material 2: Supplemental Digital Appendix 2. Outpatient Training Compared to Community based Hospitals and University Hospitals. [file 12909_2025_6670_MOESM2_ESM.pdf]
