# Supplementary material for: Optimal outpatient training for resident physicians’ general medicine in-training examination score: a cross-sectional study
Source: BMC Med Educ. 2025 Jan 11;25:49. doi: 10.1186/s12909-025-06670-5 (PMC11724509; doi:10.1186/s12909-025-06670-5)
Supplement: Supplementary file 3 — Supplementary Material 3. [file 12909_2025_6670_MOESM3_ESM.pdf]

Appendix 3: Univariate/multivariate analysis with outpatient-related GM-ITE scores as the dependent variable

	Univariable				multivariable			
		95% CI				95% CI		
	Difference	lower limit	upper limit	*p* Value	Adjusted difference	lower limit	upper limit	*p* Value
**Sex**								
Female (vs Male)	0.11	-0.103	0.313	0.321	0.110	-0.095	0.316	0.293
**Hospital location**								
Rural (vs Urban)	-0.14	-0.35	0.06	0.168	-0.21	-0.47	0.06	0.121
**Hospital type**								
Community-based hospital (vs University hospital)	0.66	0.41	0.91	<.0001	0.50	0.12	0.87	0.010
**Self-study time per day**								
None	Ref				Ref			
0-30 minutes	0.53	-0.18	1.25	0.145	0.41	-0.29	1.11	0.250
31-60 minutes	0.84	0.12	1.56	0.022	0.62	-0.09	1.32	0.086
61-90 minutes	0.83	0.08	1.59	0.030	0.60	-0.14	1.34	0.110
91 minutes or more	1.18	0.31	2.05	0.008	0.95	0.096	1.81	0.029
Duty hour								
0-59hours per week	Ref				Ref			
60-79 hours per week	0.44	0.22	0.65	<.0001	0.28	0.07	0.49	0.010
>80 hours per week	0.40	0.12	0.69	0.005	0.22	-0.07	0.51	0.136

Outpatient training period								
1 month	Ref				Ref			
2 months	-0.30	-0.56	-0.05	0.018	-0.22	-0.47	0.04	0.094
3 months or more	-0.17	-0.41	0.06	0.145	-0.21	-0.45	0.03	0.079
Outpatient training style								
block style	Ref				Ref			
parallel style	-0.15	-0.37	0.08	0.196	-0.027	-0.257	0.20	0.817
mixed style	-0.45	-0.74	-0.17	0.002	-0.323	-0.606	-0.04	0.025
New outpatient per day								
0 persons	Ref				Ref			
1 to 5 persons	0.85	0.40	1.30	0.000	0.581	0.109	1.05	0.016
6 persons or more	0.28	-0.23	0.80	0.284	0.136	-0.410	0.68	0.625
Follow-up outpatient per day								
0 persons	Ref				Ref			
1 to 5 persons	0.21	-0.02	0.44	0.074	0.137	-0.110	0.38	0.276
6 persons or more	0.02	-0.29	0.33	0.893	0.105	-0.237	0.45	0.547

CI: confidence interval.GM-ITE: General Medicine In-Training Examination. The GM-ITE score was analysed as the objective variable.
